# Room temperature passive mode-locked laser based on InAs/GaAs quantum-dot superlattice

**DOI:** 10.1186/1556-276X-7-545

**Published:** 2012-10-02

**Authors:** Mikhail Sobolev, Mikhail Buyalo, Idris Gadzhiev, Ilya Bakshaev, Yurii Zadiranov, Efim Portnoi

**Affiliations:** 1 Ioffe Physical Technical Institute, Russian Academy of Sciences, St. Petersburg, 194021, Russia

**Keywords:** Mode-locking, Laser, Polarization, Quantum dots, Superlattice, In(Ga) As/GaAs

## Abstract

Passive mode-locking is achieved in two sectional lasers with an active layer based on superlattice formed by ten layers of quantum dots. Tunnel coupling of ten layers changes the structural polarization properties: the ratio between the transverse electric and transverse magnetic polarization absorption coefficients is less by a factor of 1.8 in the entire electroluminescence spectrum range for the superlattice.

## Background

In recent years, intense efforts have been devoted to the studies of effects of tunneling coupling between electron states in semiconductor heterostructures with quantum dots (QDs), which offer much promise in the development of high-speed lasers [1], optical modulators [2], and amplifiers [3]. For optical amplifiers and modulators, it is desirable to have polarization-independent characteristics. Thus, dependencies of gain and absorption have been studied in quantum well structures [4] and QDs [5]. However, in standard uncoupled QD structures, the absorption coefficient at the lasing wavelength for transverse electric (TE)-polarized light differs by an order [2]. It is known that in structures with coupled QDs, the intensity of transverse magnetic (TM) polarization increases with the number of QD layers [2,5,6].

Direct current modulation of semiconductor lasers does not meet the needs of modern high-speed communication lines, so systems consisting of a laser and modulator are used. As more broadband alternative to the direct current modulation can be laser with integrated electro-optical modulator based on the Stark effect, high-speed performance of the Stark modulator is fundamentally limited by physical processes, namely, carrier escape from QDs and carrier removal from the *p*‐*n* junction area. Because the same processes are crucial for the passive mode-locking (PML) regime, the modulation frequency ceiling can be determined by the largest feasible PML frequency in a laser fabricated from the same structure. It should be noted that the implementation of two sectional PML lasers is technically easier than creating a high-speed modulator, because there is no need to eliminate parasitic capacitance and inductance. The modulation frequency ceiling can be determined by the largest feasible frequency of the of the PML regime in a laser fabricated from the same structure.

In this communication, we report on a room-temperature study a ten-layer system of tunnel-coupled In(Ga)As/GaAs QD. As shown in [7,8], the structure with ten tunnel-coupled layers of In(Ga)As/GaAs QDs exhibits the Wannier-Stark effect and is a quantum dot superlattice (QDSL). We have observed the EL and absorption spectra for light polarized in the plane perpendicular to the growth axis (*x* and *y*) in the same spectral range as that for light polarized along the growth direction (*z*) of the structure. No transitions involving light holes were observed in the electroluminescence and absorption spectra. The observed behavior of the measured signals allows one to conclude that the optical transitions for light polarized in the plane perpendicular to the growth axis and in the plane along the structure growth direction involve ground states of heavy holes, whose wave functions have, in addition to the *x* and *y* components, a *z* component. In this system, the ratio between the light absorption coefficients for TE and TM polarizations is close to 1 in contrast to structures with unbounded QDs, where the ratio is about 10. This makes it a promising structure for optical polarization-independent modulators used in fiber-optic communication lines (FOLs). Two sectional PML laser diodes with an absorbing section acting as modulator were made from the SLQD structure. It shows the fundamental possibility of implementing a laser and modulator in a monolithically integrated design.

## Methods

**Figure 1:**
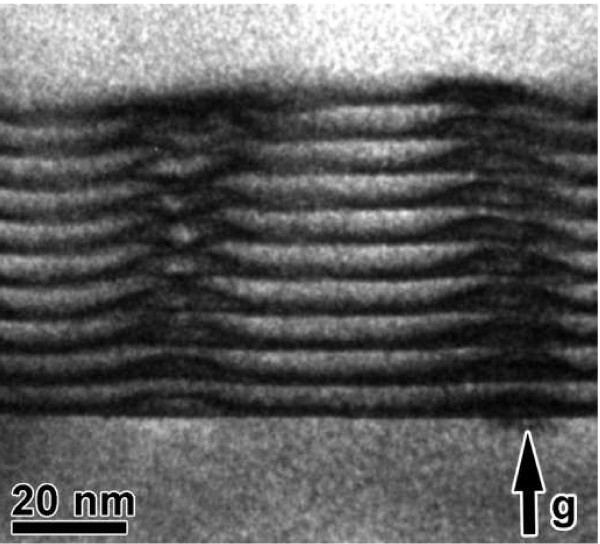
**Transmission electron micrographs of the cross section of the sample.** The sample has ten InAs QD layers and iswith a GaAs spacer layer 6.0-nm thick between them.

Laser structures were grown by molecular beam epitaxy on *n*^*+*^ -GaAs (001) substrate and are similar to the structure described in [6,8]. The structure consisted of an *n*-doped bottom Al_0.35_ Ga_0.75_As layer with a thickness of 1.5 μm, a waveguide undoped GaAs layer with a thickness of 480 nm containing ten layers of In(Ga)As QD, a *p*-doped upper Al_0.35_ Ga_0.75_As layer with a thickness of 1.5 μm, and a *p*^*+*^ -doped contact GaAs layer. QD ensembles were grown ten times by InAs 2.3 monolayer deposition with GaAs barrier layers with a thickness of 6 nm between QD layers. Thus, layers of self-organized In(Ga)As QDs were built into the central part of the undoped GaAs matrix. The refractive index of the upper and lower Al_0.35_ Ga_0.75_As layers differed from that of the central layer, which confined light within the central part of the undoped SLQD-containing region. The vertical alignment of QDs was observed by transmission electron microscopy (see Figure 1) [6,8] .

Two sectional lasers were fabricated from SLQD structures. Standard lithography techniques were used to make a 5-Âµm strip forming a single-mode waveguide. The cavity length was 3.5 mm, the absorber length was 10% of the cavity length, and the sections were electrically isolated by the gap in the contact. This laser design is in fact standard and is described in various publications [2,8-10] but differs from them in that the active layer is SLQD, formed by ten QD layers and thin barrier layers between them. The devices were mounted on a copper heat sink; all measurements were performed at room temperature.

**Figure 2:**
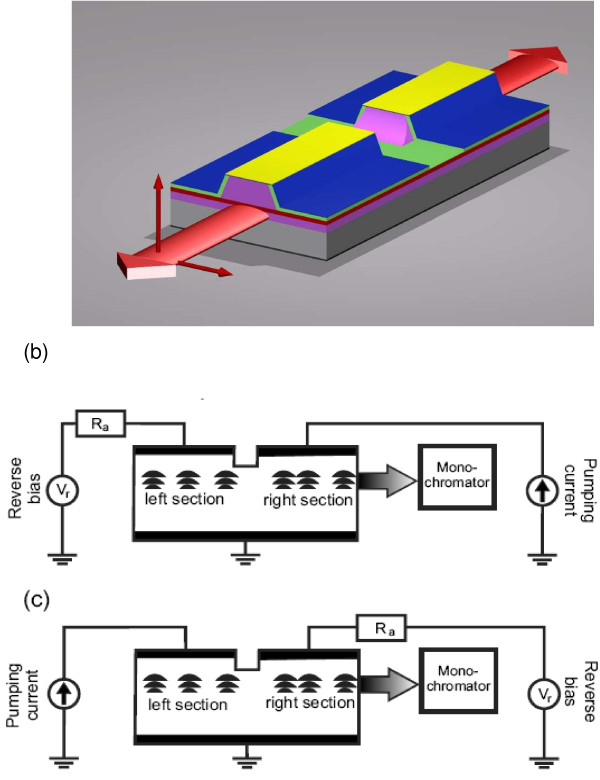
**Schematic of a double-section laser design and the experimental setup for SLQD absorption measurements. (a)** Schematic of a double-section laser design with amplifying and absorbing sections, used to measure the electroluminescence, and absorption spectra for two light polarization directions: in the plane perpendicular to the growth axis (⊥) (*x*–*y* plane) and along the growth direction (||) (*z* axis) of the structure. **(b)** and **(c)** The experimental setup for SLQD absorption measurements: **(b)** direct detection of electroluminescence spectra and **(c)** detection of electroluminescence passed through absorbing section.

Absorption measurements were provided as described in [8,9] using this device. The experimental setup is shown on Figure 2b,c. A sample with two equal sections was used. The emission in waveguide was excited by the current injection in one of the sections; the pumping current is far below the threshold current. On the first stage (Figure 2b), the emission spectrum (I_0_) from the closest section to the monochromator section (the right setion on Figure 2b) is measured, nothing is applied to the other section. Thus, the spectrum of source light is obtained. Next, the closest section is reverse-biased, and the other section is pumped with the same current as the right section in the first stage (Figure 2c). In this waveguided setup, radiation from the left section penetrates into the right section almost without loss, then experiences partial absorption by SLQDs in the right section, and reaches measuring setup through low-reflectance facet. Hence, the emission spectra of passed light through the absorption section (*I*_0_·e^-k(V)l^) is obtained. Since both sections have the same length and the optical scheme of the experimental setup was not changed, one can assume that the intensity of the emission reaching the absorber section on the second stage is approximately equal to the intensity measured on the first stage. This allows the derivation of the SLQD absorption spectra in absolute values.

PML investigation was under pulsed current injection (pulse duration 1 μs) and direct current (DC) reverse bias. An autocorrelation setup based on a Michelson interferometer was used for pulse duration measurements, controlled by an oscilloscope with a 50-GHz bandwidth, an electrical spectrum analyzer with a 22-GHz bandwidth, and a 20-GHz photodetector. The devices were mounted to copper heat sink; all measurements were done at room temperature.

## Results and discussion

**Figure 3:**
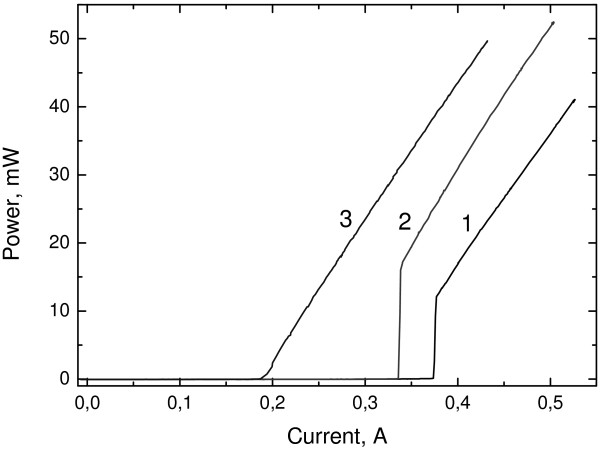
**Watt-ampere characteristics of the laser at various currents in absorber section, the optical power from one mirror. ** 1 is I_r_ = 0mA and I_th_ = 313 mA; 2 is I_r_ = 0.08 mA and I_th_ = 262 mA; 3 is I_r_ = 15 mA and I_th_ = 186 mA.

The devices were pumped by DC in light-current and absorption experiments. A clear, rigid switching-on effect is observed, which is eliminated only when significant forward current *I*_*r*_*is* applied to the absorber section (Figure 3). Threshold current *I*_*th*_ decreases with forward bias applied to the absorber section, increasing with a minor change in differential efficiency. Rigid switching on is related to the optical bistability effect induced by absorber bleaching because the carrier escape speed is not high enough. This rigid switching-on effect is a characteristic phenomenon of two sectional QD lasers with PML.

**Figure 4:**
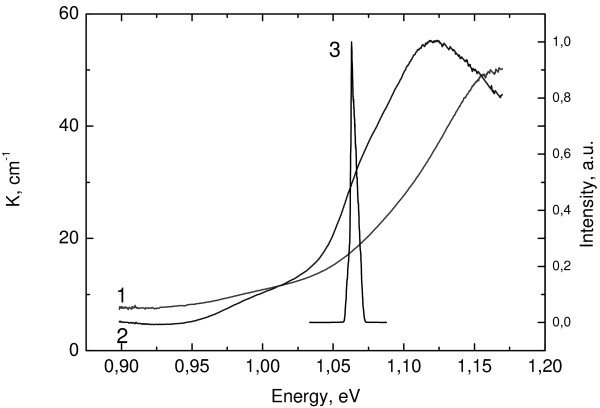
**The absorption laser spectra.** The absorption spectra for TM and TE mode (lines 1 and 2) and of laser spectra in PML regime (line 3).

Figure 4 shows the light absorption spectra for the emission and absorption sections with an In(Ga)As/GaAs SLQD. The spectra were measured for two polarization directions: in the plane perpendicular to the growth axis (*x* and *y* planes) and along the structure’s growth direction (*z* axis). Commonly, by these polarization directions are meant the TE and TM modes, respectively.

At the lasing wavelength in the PML regime, the absorption coefficient for TM-polarized light is only 1.6 times smaller than that for TE polarization (Figure 4). The maximum ratio of absorption coefficients reaches 1.8 and at energies less than 1.012 eV and more than 1.156 eV; absorption for TM polarization is more (Figure 4, lines *1* and *2*). The electroluminescence spectrum width in the laser structure at a current density of *J* = 0.3*J*_*th*_ is about 130 nm, where *J*_*th*_ is the threshold current density. It is due to two factors: the QD size dispersion and energy level splitting with QD coupling. The absorption coefficient front is about 90 nm, which is comparable to the electroluminescence spectrum width.

**Figure 5:**
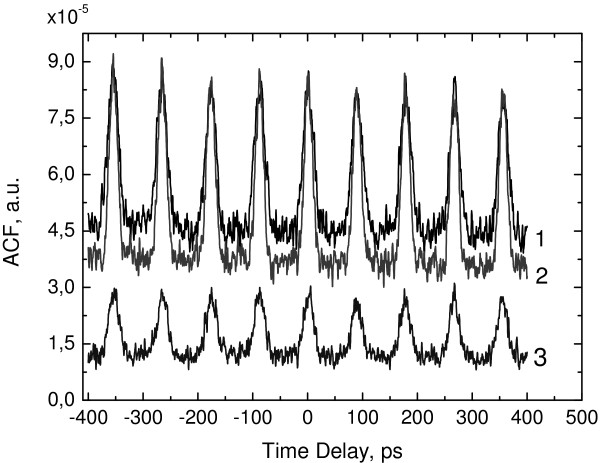
**Autocorrelation functions of the laser at different reverse bias: 1 - V**_**r**_ **=-1V, 2 - V**_**r **_**=-2V, 3 - V**_**r **_**=-3V.**

Lasing spectra lay in the range 1,160 to 1,170 nm (Figure 4, line *3*) and shift to the longwave region with reverse bias increase. The FWHM of the spectrum at *V*_*r*_ = −2 V is 5.2 nm. PML was observed in wide injection current range at reverse bias from −1 to −3 V (Figure 4) with a repetition frequency of 12.5 GHz. At low reverse biases the carrier lifetime in the absorber section τ_*abs*_ is much more than the roundtrip time τ_*R*_, so there is no mode-locking. With reverse bias increasing the carrier escape rate grows up and τ_*abs*_ becomes comparable with the round-trip time, so short pulse observation becomes possible. The smallest pulse duration is achieved at injection currents near the threshold and reverse bias at −2 V. The pulse width at half maximum was derived from the measured autocorrelation function (Figure 5), and the assumption of the Gaussian pulse profile is 10 ps. This gives a time-bandwidth product value of about 10. Such large value is due to the high injection current and the fact that measurements were done in pulsed mode. An increase in reverse bias leads to PML collapse and the laser emits in the cw mode. It is due to the shift of laser spectra to the longer wave-length region where absorption modulation of saturated and unsaturated states of the saturable absorber is not enough for mode-locking.

## Conclusions

In conclusion, based on a structure containing ten layers of coupled QDs, two sectional lasers were created in which PML realization needs a rather small reverse bias on the absorber section. The absorption value both for TM and TE polarizations exceeds 50 cm^–1^, which is sufficient for modulators used in FOLs. In contrast to the structure with uncoupled QDs, where TM polarization can be neglected, the luminescence intensity and absorption coefficients for TE and TM polarizations in SLQD are comparable.

## Competing interests

The authors declare that they have no competing interests.

## Authors’ contributions

MS supervised the project and provided laser structures and drafted the manuscript. MB carried out experimental studies, analyzed and interpreted the data and participated in drafting of the manuscript. IM carried out samples characterization and experimental studies, analyzed and interpreted the data. IO carried out absorption measurements. YuZ fabricated the samples from laser structures. EP provided critical review and final approval of the article. All authors discussed the results and implications and commented on the manuscript at all stages. All authors read and approved the final manuscript.
